# Socio-economic position, area-level deprivation and gradients in cancer incidence: England and Wales, 1971–2016

**DOI:** 10.1186/s12889-025-21875-5

**Published:** 2025-02-24

**Authors:** Robert A. Hiatt, Wei Xun, Eduardo J. Santiago-Rodríguez, Jitka Pikhartova, Nicola Shelton

**Affiliations:** 1 University of California San Francisco, 550 16th Street, 2nd Floor, San Francisco, CA 94158 USA; 2 University College London, London, England UK

**Keywords:** Cancer incidence, Social gradients, Longitudinal study, England and Wales

## Abstract

**Background:**

Social gradients for cancer mortality and survival have been reported but are less clear for cancer incidence where social factors external to health care systems are likely to be of more etiologic importance.

**Methods:**

We examined social gradients in cancer incidence using data from the Office for National Statistics Longitudinal Study (ONS-LS), which selects an approximately 1.1% representative sample of the population of England and Wales. Data were analyzed for each successive ten-year census period from 1971–2011 with outcome data to 2016, the latest date available. Socioeconomic position of individuals was assessed using the National Statistics Socio-economic classification (NS-SEC). Areal level deprivation was measured using deciles of the Townsend Index. Cancer outcomes from the National Cancer Intelligence Network linked to the ONS-LS were examined for all cancers, and more common individual cancer sites. We used logistic regression to generate odds ratios to estimate the risk of a first incident cancer within each follow-up period.

**Results:**

The 1971 ONS-LS census sample population initially comprised 257,803 individuals updated each census; and by 2016 137,755 incident cancer cases. Social gradients in cancer incidence were present for individual cancer sites of lung, stomach, and cervix for both individual and areal measures of socioeconomic standing with the least advantaged having higher incidence rates. Reverse gradients were present for prostate and breast cancers. The relationship of SES to increased cancer incidence for these common cancers is consistent with prior literature, but the striking gradients in these relationships reveal the strong association of SES factors with increasing social disadvantage for these cancers.

**Conclusion:**

The findings demonstrate the importance of socioeconomic position in the incidence of some common cancers prior to diagnosis and treatment and reinforces the need for further research to address the contribution of upstream social determinants in the etiology of cancer.

**Supplementary Information:**

The online version contains supplementary material available at 10.1186/s12889-025-21875-5.

## Introduction

That there are social determinants of health has now become well engrained in the population health literature and a stimulus for research and action based on underlying aspects of social conditions [1]. For cancer, although most recent advances have been in the areas of molecular biology and therapeutics, a focus on inequities in outcomes has also advanced the field of cancer population health [2]. However, mechanisms that explain observed inequalities have been more elusive. Social gradients in cancer have been documented in some studies, although they are not as strong as those seen for overall mortality or cardiovascular disease [3, 4]. But why should a set of diseases like cancer, which are fundamentally caused by mutations in DNA, be associated with social position? Where gradients exist, what does it augur for the cancer research agenda and possible actions at the policy level?

The evidence for social gradients in cancer come from several sources focused on mortality and survival outcomes. In general, cancer mortality demonstrates a social gradient with the less socially advantaged having, with some exceptions, the highest death rates from cancer [1, 5–10]. However, cancer incidence better reflects the influence of the social environment on cancer etiology without the contribution of health care access and quality that come into play with survival and mortality [11, 12]. Social factors influencing cancer incidence are less well studied. Tobacco use is a major cause of cancer and is more prevalent in recent decades in lower socioeconomic position (SEP) groups [13]. However, tobacco use gradation by SEP does not explain the entire cancer social gradient and is not strongly related to most common cancers except lung [9].

Our intent in this research was to further explore the relationship between socioeconomic position and cancer incidence by analyzing existing longitudinal data from the United Kingdom from two sources: 1) the Office for National Statistics Longitudinal Study (ONS-LS), and 2) linked cancer registry outcomes in the National Cancer Intelligence Network (NCIN).

## Methods

### Study participants

For this longitudinal observational study, we used existing data from the ONS-LS, which links over 1.5 million census records and life events data from official registries of deaths, cancer incidence, and births in a representative sample of approximate 1.1% of the population of England and Wales. The ONS-LS now contains over 500,000 individuals from each of five decennial censuses 1971 to 2011 (data is not yet available for the 2021 census). It uses a dynamic sampling method wherein new members are added to the data from qualifying new births, immigration and succeeding censuses, so that starting at each census it is possible to construct similar-sized but distinct cohorts (more information about the study can be found in the Cohort Profile) [14].

The ONS has not sought the consent of people included in the LS. The need to consent to participation was unnecessary according to regulations of the UK Statistics Authority's Research Accreditation Panel that provides oversight of the framework that is used to accredit research projects, researchers and processing environments under the Digital Economy Act 2017 (DEA) and the Approved Researcher gateway in the Statistics and Registration Services Act 2007 (SRSA). https://uksa.statisticsauthority.gov.uk/digitaleconomyact-research-statistics/research-accreditation-panel/

### Cohort and decade sample populations

**Fig. 1:**
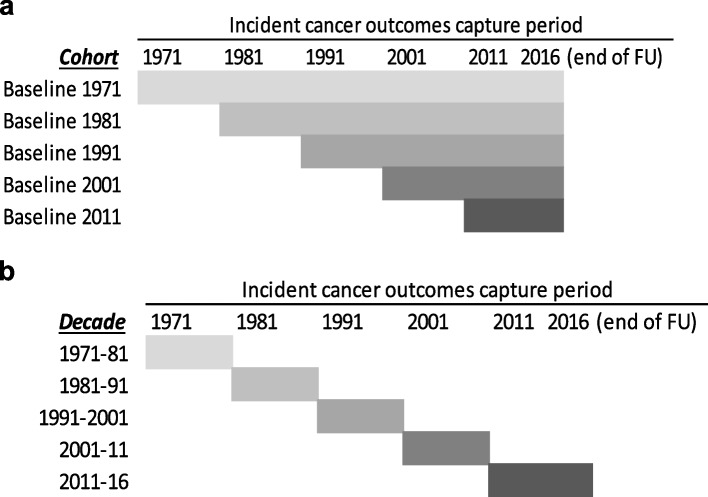
Office for National Statistics Longitudinal Study (ONS-LS) data set-ups: **a**- Cohorts, **b**- Decades

We utilized ONS-LS Census data to construct five cohorts starting in 1971 and each decadal census that followed (i.e., 1981, 1991, 2001, and 2011) and separately the five decadal 10-year follow-up cohorts. For the baseline cohort (Fig. 1-a), we selected all LS sample members present at each of the decennial census 1971 to 2011 and followed the sample members for cancer incidence until the end of the follow-up period (31st Dec 2016 the latest data available). While for decade samples (Fig. 1-b), we again selected sampled members from each of the five censuses but followed their cancer incidence for the 10 years until the next census except for the 2011 which was followed for five years. This allowed us to examine changes in cancer incidence gradients over 45 years for four consecutive ten-year periods independent of the data from earlier decades.

### Outcomes

Our principal outcome was new cancer registrations, using the ICD codes current at the time to record the type and site of the cancers. The cancer incidence information in the ONS LS [15, 16], comes from the National Cancer Registration Service [17], which includes all malignant neoplasms and the reticuloses (C00 to C97 inclusive of the Tenth Revision to the International Statistical Classification of Diseases and Related Health Problems (ICD10)). First primary malignant cancer diagnosis between 26th April 1971 and 31st December 2016 were recorded. We further grouped all cancers captured in the dataset into two outcome sets: 1: all cancers; 2: all cancers excluding smoking-related cancers (i.e., lung and oral).

### Exposures

#### The National Statistics Socio-economic classification (NS-SEC)

The exposures in our study were measures of individual social position based on occupation, formerly Registrar General’s Social Class introduced in 1913. All SEP information in the ONS-LS was derived using occupational information collected at each census. Social class was replaced by the National Statistics Socio-economic classification (NS-SEC) from 2001 onwards. The NS-SEC was designed to explicitly measure occupational relationships and conditions [15, 18] by distinguishing individuals based on their ‘employment relations’, ranging from people who provide services in return for compensation to those who provide labour in return for a wage [19]. The current study uses an ordinal three-class schema [20] classified as: 1. Managerial and professional occupations; 2. Intermediate occupations (set as reference); 3. Routine and manual occupations. Individuals without an occupation were excluded from the analytic samples meaning women and those who were economically inactive are underrepresented in the earlier cohorts. The high degree of heterogeneity in this group would make any results difficult to interpret so we judged that it was best to exclude them. NS-SEC was assigned retrospectively to the 1971–1991 cohorts.

#### Area-level deprivation

We also examined an area-level deprivation index, the Townsend Score [21, 22] calculated for England and Wales at ward and Lower Super Output Area levels [23] for the census years 1971 to 2011. The Townsend Score strives to represent relative material deprivation and encompasses multiple dimensions including census information regarding male labour market participation, car access, household overcrowding (defined as more than 1 person per room) and housing tenure. The Townsend Score has consistently been associated with health outcomes at small geographical levels [24]. We had no data on cancer risk behaviours including smoking status.

#### Analytic Methods

We used logistic regression to generate odds ratios that estimate the probability of first incident cancer outcomes within each of the follow-up periods between levels of baseline SEP, by sex. We sought to observe gradients in cancer outcomes by individual and group measures of SEP wherein incidence was higher (or lower) for each level of SEP. A social gradient is one where the population is categorized and then ranked by a measure of socioeconomic status or position [1]. This is a descriptive study and no particular hypothesis was being tested. For the individual level NS-SEC, we applied models for each the baseline cohort and each of the five decadal cohorts controlling for the following baseline characteristics: linear age and age-squared, education level (none/none stated, below degree; degree or higher), marital status (married/civil partnership; widowed; divorced/separated; single/never married) and grouped country of birth. We repeated the same models for area-level deprivation using the Townsend Score, and finally we simultaneously adjusted for NS-SEC and Townsend Score. We ran sex-specific analyses for prostate, bladder and stomach cancers in men, and breast, uterus, cervical and ovarian cancers in women. We present odds ratios and 95% confidence intervals.

## Results

The sample population in 1971 was comprised of 257,803 participants, 90,757 (35%) women and 167046 (65%) men. In subsequent years the total number in the study population varied by decade with new participants added due to births and immigration, and participants lost due to death, emigration, and follow-up. The numbers of men sampled was similar until 2011 when it increased to 192,752. The numbers of women increased substantially starting in 1991 and reached 202,291 in 2011 consistent with the number of women entering the workforce and thus classifiable by the NS-SEC.

A total of 137,755 cancer cases were included in the study, 72,837 (53%) women and 64,918 (47%) men. Most cases (69%) were diagnosed at ≥ 60 years of age, although women were generally diagnosed at younger ages, with 38% having a diagnosis before 60 years (versus 23% of men). The distribution of cases by age at diagnosis and sex up to 2016 is presented in Supplemental Table 1.

The most common cancer sites that comprised 38% of all cases and among men were lung (16%), prostate (14%), colorectal (10%), bladder (4%) and stomach (4%). Among women, breast (20%), colorectal (8%), lung (7%), ovary (3%) and uterus (2%) occupied the top five cancer sites. A complete distribution of the top 20 cancer sites, overall and by sex, is presented in Supplemental Table 2.

### NS-SEC

**Fig. 2:**
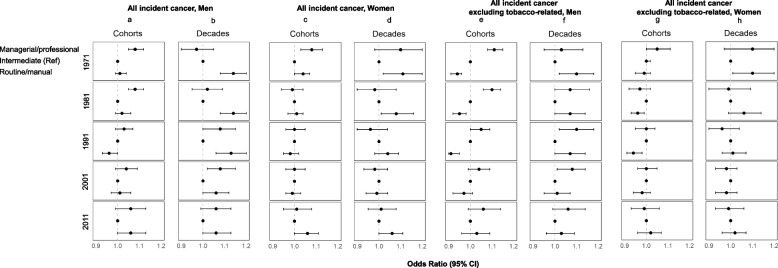
Cancer incidence in the Office for National Statistics Longitudinal Study (ONS-LS) according to the National Statistics Socio-economic classification (NS-SEC), 1971–2016. Overall cancer incidence for men and women (Panels **a**-**d**), all cancer with tobacco-related cancers excluded for men and women (Panels **e**–**h**). Analyses were adjusted for age, education level, marital status and country of birth group

The association between NS-SEC and all incident cancers combined varied by sex (Fig. 2). In the earlier first three decades of baseline cohort follow-up men in managerial and professional occupations had higher incidence of cancer than those in intermediate or routine and manual occupations (Panel 2a). In the evaluation by decades, however, men employed in managerial and professional roles entering the study during the first three decades had a lower incidence of cancer than men reporting routine and manual occupations although this relationship was not sustained in later decades (Panel 2b). No clear pattern was identified among women (Panel 2c). There was a gradient in the second and third decades for women where those employed in managerial and professional roles had lower incidence, but this pattern was not consistent in all decades (Panel 2d). In the additional analyses grouping all cases and excluding tobacco-related cancers (Panels 2e-h) gradients for men were more marked with managerial and professions having higher rates.

**Fig. 3:**
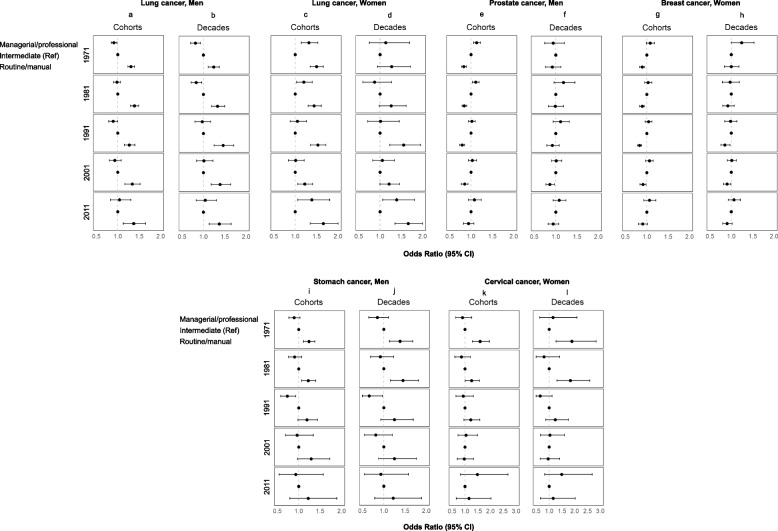
Cancer incidence in the Office for National Statistics Longitudinal Study (ONS-LS) according to the National Statistics Socio-economic classification (NS-SEC), 1971–2016. Cancer sites: Lung for men and women (Panels **a**-**d**), Prostate (Panels **e**–**f**), Breast (Panels g-h), Stomach in men (Panels **i**-**j**), and Cervix (Panels **k**-**l**). Analyses were adjusted for age, education level, marital status and country of birth group

In the assessment of individual cancer sites (Fig. 3), strong inverse gradients between NS-SEC and lung cancer were observed among men both by cohorts and decades, with those reporting routine and manual occupations having higher incidence of lung cancer than their counterparts (Panels 3a and 3b). Among women, those with routine and manual occupations also had higher incidence of lung cancer, but gradients were less consistent (Panels 3c and 3d).

Direct gradients between NS-SEC and both prostate and breast cancer were observed in the analyses of cohorts (Panels 3e and 3 g). Individuals in managerial and professional occupations had the highest incidence of these cancers. This gradient in individual decades for breast cancer was attenuated in the four most recent decades (Panel 3 h).

Socioeconomic gradients between NS-SEC and stomach cancer in men and cervical cancer in women were similar to the patterns with lung cancer. In the first two cohorts, people in routine and manual occupations had higher incidence of these cancers than those in intermediate or managerial/professional occupations (Panels 3i and 3 k). People in managerial and professional occupations had the lowest incidence, but gradients became attenuated in more recent time periods for women with cervical cancer. Similar results were observed by decades (Panels 3j and 3 l). No clear gradients were observed for the other common cancer sites, including colon and rectum, bladder in men, ovary, and uterus (Supplemental Fig. 1).

### Townsend deprivation index

**Fig. 4:**
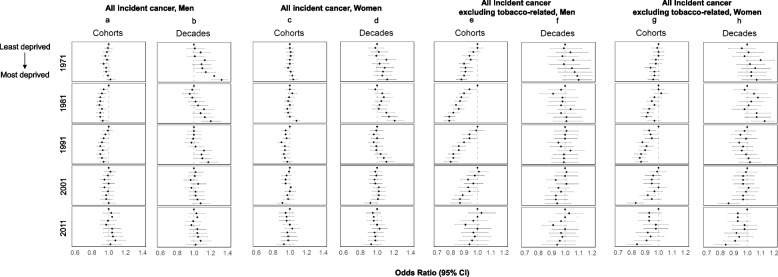
Cancer incidence in the Office for National Statistics Longitudinal Study (ONS-LS) according to the Townsend Deprivation Index, 1971–2016. Overall cancer incidence for men and women (Panels **a**-**d**), all cancer with tobacco-related cancers excluded for men and women (Panels **e**–**h**), and all cancer excluding screening-related cancers for men and women (Panels **i**-**l**). Analyses were adjusted for age, education level, marital status and country of birth group

For areal deprivation as measured by the Townsend Score, we found mixed results for overall cancer incidence in men (Fig. 4) with the suggestion of an inverse relationship between deprivation and cancer incidence (Panel 4a). Those residing in more deprived areas had higher cancer incidence up until 2001 but not thereafter (Panel 4b). Among women, no clear patterns were identified in the longitudinal cohort (Panel 4c), but a higher incidence was observed in women residing in the most deprived areas during the first three decades (Panel 4d). In the set of additional analyses grouping all cases and excluding tobacco-related cancers, findings were consistent with the patterns previously described for NS-SEC where both men and women from less deprived areas had higher cancer incidence (Panels 4e-4 h).

**Fig. 5:**
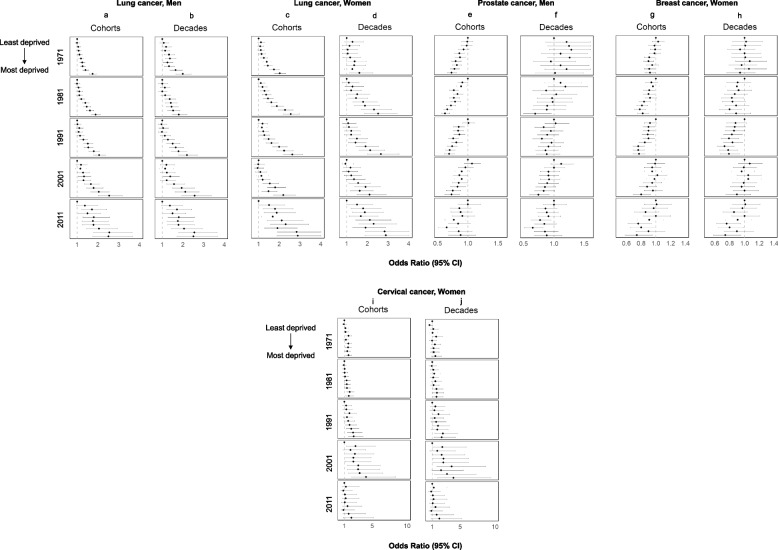
Cancer incidence in the Office for National Statistics Longitudinal Study (ONS-LS) according to the Townsend Deprivation Index, 1971–2016. Cancer sites: Lung for men and women (Panels **a**-**d**), Prostate (Panels **e**–**f**), Breast (Panels **g**-**h**), and Cervix (Panels **i**-**j**). Analyses were adjusted for age, education level, marital status and country of birth group

In the evaluation of individual cancer sites (Fig. 5), there were clear social gradients for men and women residing in more deprived areas having higher lung cancer incidence in all cohorts and decades (Panels 5a-5d). For prostate and breast cancer, inverse associations in gradients were observed between deprivation and incidence in most cohorts (Panels 5e and 5 g). For cervical cancer, women residing in more deprived areas had higher incidence in clear but less marked gradients compared to the more common cancers just described (Panels 5i and 5j). We did not find evidence of associations between deprivation and incidence of cancer for other sites (Supplemental Fig. 2). In analyses of the area level with the Townsend Score adjusted for individual NS-SEC we saw similar results to those where NS-SEC was not included (Supplemental Figs. 3-5).

## Discussion

We set out to explore evidence for social gradients in cancer incidence in the large ONS-LS dataset for England and Wales for the 45-year span from 1971 to 2016. Our hypothesis was that if social gradients existed for incidence as distinct from survival or mortality, it would suggest that the social context, exclusive of the influence of access to and quality of medical care, might contribute to the etiology of those cancers and thus point to directions for interventions at a population or policy level. We were interested in incidence as opposed to cancer survival or mortality since social factors driving incidence are more likely to be associated with the social and behavioral environment and not with characteristics of treatment and cancer care that have their impact once individuals enter healthcare systems.

The ONS-LS allowed us the unusual opportunity to examine these questions in a large population-based sample as if we were tracking a cohort over a 45-year period as well as smaller cohorts defined by 10-year census cycles. This dual perspective provided information about the associations of social position in a retrospectively constructed cohort over a long period of time as well as the same associations in ten-year increments presumably as they changed with social and environmental conditions. This allowed us to observe the consistency of the gradients over time.

As expected, the relationship of social class as measured by individual NS-SEC varied by cancer sites and over time. Results for the overall baseline cohort for 1971suggested that men in the managerial and professional categories actually had a higher incidence of cancer whereas in the individual decades they had the lowest incidence. One possible explanation for this paradox is that with longer follow-up mortality was probably greater in men with lower SEP leaving more high SEP individuals in the cohort. As they aged up over the 45-year period of follow-up men in the highest NS-SEC categories developed more cancer with advanced age.

There were some striking gradients observed for specific cancers sites, namely lung, stomach, and cervix where we observed that individuals of lower social class experiencing higher rates of new cancers. Contrarywise for breast and prostate higher SEP individuals experiencing higher rates in gradients. These relationships are consistent with the literature and well established for these sites apart from prostate for which the relationship to class has not been consistent [25, 26]. PSA screening was not systematically studied until the late 1990s in the UK so would not likely have contributed in the 1971–1991 cohorts [27]. However, the association of social class and prostate cancer incidence may be due to increased awareness of the disease, more frequent medical visits and opportunities to be tested [28]. The absence of a gradient for colorectal cancer was unexpected, since inverse social gradients in incidence for this cancer have been observed in the U.S. and other high-income countries presumably due to increase surveillance and screening [29, 30]. A recent study in the UK using the same ONS data also found no relationship with colorectal cancer incidence but did for survival and mortality with a graded area deprivation index suggesting that social status had more to do with access and quality of medical care than etiology [31]. We did not observe social gradients in other less common cancers.

On the possibility that overall gradients would be observed in subgroups formed by known risk factors we looked at all cancers except tobacco-related cancers (i.e., lung, oral)) but no clear gradients were observed with individual social class. Areal deprivation did show some clear social gradients with cancer incidence that were like those observed for NS-SEC.

The relationship of SEP and cancer incidence has been studied by others [5, 8, 30, 31] and gradients have been demonstrated for specific cancer sites but not for cancer overall consistent with our findings. Clearly the heterogeneity of 'cancer' as a single entity rather than a collection of tumors of different sites makes overall conclusions difficult. For specific sites, however, we, like others, have demonstrated inverse relationship with lung, cervix, and stomach as well as direct relationships with breast. The data for colorectal and prostate, are less consistent. Without behavioural data in the ONS-LS we cannot evaluate the effect of tobacco directly but the inverse gradient with lung cancer follows existing knowledge about the etiologic effects of tobacco use by social class and the known impact of marketing practices by the tobacco industry[6]. For cervical cancer incidence the gradient with social class and deprivation measures is consistent with behavioral and social practices associated with increased HPV exposure[32]. The direct relationship of social class with breast cancer is also well known and attributed primarily to reproductive practices associated class such as lower age at menarche, higher age at menopause, later age at first pregnancy, and lower parity and to a certain extent to alcohol consumption (33).

The strengths of our study include its large sample size with data on cancer incidence for all the common cancers over a 45-year period. We were also able to examine the social gradients that existed in 10-year increments over that 45-year period which illustrated changes in those gradients with time likely due to changes in societal patterns of employment and normative behaviors. Where social gradients exist it suggests that socioeconomic factors associated with level of the gradient and not something to do with the biology of cancer were in play. The main limitation, as mentioned, was that we had no behavioral data on tobacco use, dietary practices, physical activity, or cancer screening practices. Also, SEP as measured by the NS-SEC does not directly take into account education and income as do other measures of SES, although these factors are reasonable reflected in occupational position. There is a possibility that different life expectancies over the duration of the study could have influenced the results, but we did not conduct a separate age-period-cohort analysis. Finally, the Townsend Index, although a widely used measures of areal deprivation, does not adequately capture any psychosocial stress component associated with grade of employment or living in areas of relative deprivation.

## Conclusion

We observed social gradients for specific cancer sites that were consistent with known categorical risk factors for these cancers. The variation by census decade may be compatible with changes in the social environment (e.g., labour practices for women) over the decades. The findings reinforce the notion that for cancer sites where behaviors are known to play a role in their etiology, social gradients in incidence are observed. This supports the idea that the etiology of these cancers are at least partially driven by social circumstances and suggests that more emphasis needs to be placed on policy interventions to address upstream social determinants of cancer.

## Supplementary Information

Supplementary Material 1.

Supplementary Material 2.

Supplementary Material 3.

Supplementary Material 4.

Supplementary Material 5.

Supplementary Material 6.

## Data Availability

The raw ONS LS and linked cancer incidence data are protected and are not openly available due to data privacy laws. Researchers wishing to use ONS LS data need to successfully complete an Accredited Researcher application and also need their project to be approved by the UK Statistics Authority's Research Accreditation Panel. Data extracts can only be accessed via the Secure Research Service and from within the UK. https://www.ons.gov.uk/aboutus/whatwedo/paidservices/longitudinalstudyls#privacy-and-data-protection ONS Longitudinal Study ONS, 2024.

## Abbreviations

DNA: Deoxyribonucleic acid
HPV: Human papillomavirus
ICD: International Classification of Diseases
NCIN: National Cancer Intelligence Network
NS-SEC: National Statistics Socio-economic classification
ONS-LS: Office for National Statistics Longitudinal Study
PSA: Prostate specific antigen
SEP: Socioeconomic position
