# Supplementary material for: Socio-economic position, area-level deprivation and gradients in cancer incidence: England and Wales, 1971–2016
Source: BMC Public Health. 2025 Feb 24;25:741. doi: 10.1186/s12889-025-21875-5 (PMC11849355; doi:10.1186/s12889-025-21875-5)
Supplement: Supplementary file 4 — Supplementary Material 4. [file 12889_2025_21875_MOESM4_ESM.pdf]

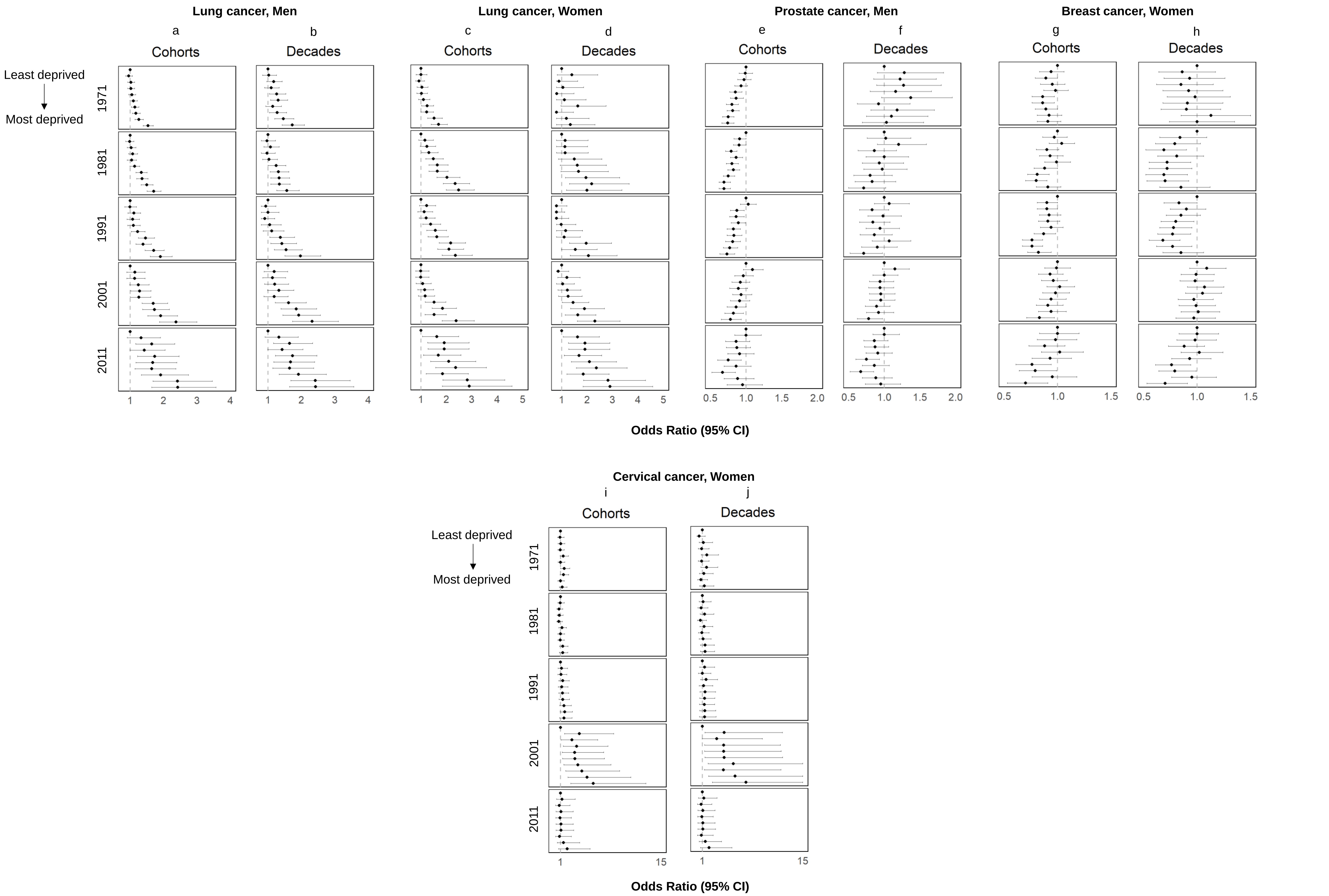

Supplemental Figure 4. Cancer incidence in the Office for National Statistics Longitudinal Study (ONS-LS) according to the Townsend Deprivation Index and adjusting for the National Statistics Socio-economic classification (NS-SEC), 1971-2016. Cancer sites: Lung for men and women (Panels a-d), Prostate (Panels e-f), Breast (Panels g-h), and Cervix (Panels i-j). Analyses were also adjusted for age, education level, marital status and country of birth group.

Source: ONS LS
