# Supplementary material for: Socio-economic position, area-level deprivation and gradients in cancer incidence: England and Wales, 1971–2016
Source: BMC Public Health. 2025 Feb 24;25:741. doi: 10.1186/s12889-025-21875-5 (PMC11849355; doi:10.1186/s12889-025-21875-5)
Supplement: Supplementary file 6 — Supplementary Material 6. [file 12889_2025_21875_MOESM6_ESM.pdf]

Submission ID 4762b0e7-b97c-483d-b34d-e118ec379a7d

Socio-economic Position, Area-Level Deprivation and Gradients in Cancer Incidence: England and Wales, 1971-2016

Supplemental Figure information

Supplemental Figure 1. Cancer incidence in the Office for National Statistics Longitudinal Study (ONS-LS) according to the National Statistics Socio-economic classification (NS-SEC), 1971-2016. Cancer sites: Colon and rectum for men and women (Panels a-d), Bladder in men (Panels e-f), Ovary (Panels g-h), and Uterus (Panels i-j). Analyses were adjusted for age, education level, marital status and country of birth group.

Supplemental Figure 2. Cancer incidence in the Office for National Statistics Longitudinal Study (ONS-LS) according to the Townsend Deprivation Index, 1971-2016. Cancer sites: Colon and rectum for men and women (Panels a-d), Bladder in men (Panels e-f), Ovary (Panels g-h), and Uterus (Panels i-j). Analyses were adjusted for age, education level, marital status and country of birth group.

Supplemental Figure 3. Cancer incidence in the Office for National Statistics Longitudinal Study (ONS-LS) according to the Townsend Deprivation Index and adjusting for the National Statistics Socio-economic classification (NS-SEC), 1971-2016. Overall cancer incidence for men and women (Panels a-d), all cancer with tobacco-related cancers excluded for men and women (Panels e-h), and all

cancer excluding screening-related cancers for men and women (Panels i-l). Analyses were also adjusted for age, education level, marital status and country of birth group.

Supplemental Figure 4. Cancer incidence in the Office for National Statistics Longitudinal Study (ONS-LS) according to the Townsend Deprivation Index and adjusting for the National Statistics Socio-economic classification (NS-SEC), 1971-2016. Cancer sites: Lung for men and women (Panels a-d), Prostate (Panels e-f), Breast (Panels g-h), and Cervix (Panels i-j). Analyses were also adjusted for age, education level, marital status and country of birth group.

Supplemental Figure 5. Cancer incidence in the Office for National Statistics Longitudinal Study (ONS-LS) according to the Townsend Deprivation Index and adjusting for the National Statistics Socio-economic classification (NS-SEC), 1971-2016. Cancer sites: Colon and rectum for men and women (Panels a-d), Bladder in men (Panels e-f), Ovary (Panels g-h), and Uterus (Panels i-j). Analyses were also adjusted for age, education level, marital status and country of birth group.
